# Advancing the pragmatic measurement of sustainment: a narrative review of measures

**DOI:** 10.1186/s43058-020-00068-8

**Published:** 2020-09-17

**Authors:** Joanna C. Moullin, Marisa Sklar, Amy Green, Kelsey S. Dickson, Nicole A. Stadnick, Kendal Reeder, Gregory A. Aarons

**Affiliations:** 1 Faculty of Health Sciences, School of Pharmacy and Biomedical Sciences, Curtin University, Kent Street, Bentley, Perth, Western Australia 6102 Australia; 2 Child and Adolescent Services Research Center, 3665 Kearny Villa Rd., Suite 200N, San Diego, CA 92123 USA; 3 Department of Psychiatry, University of California San Diego, 9500 Gilman Drive (0812), La Jolla, CA 92093-0812 USA; 4 UC San Diego Dissemination and Implementation Science Center (UC San Diego DISC), Altman Clinical and Translational Research Institute, 9500 Gilman Drive (0990), La Jolla, CA 92093-0990 USA; 5 The Trevor Project, PO Box 69232, West Hollywood, CA 90069 USA; 6 San Diego State University, 5500 Campanile Drive, San Diego, CA 92182 USA

**Keywords:** Sustainment, Sustainability, Maintenance, Implementation, Measurement, Scale, Psychometric properties, Knowledge Translation

## Abstract

**Background:**

Sustainment, an outcome indicating an intervention continues to be implemented over time, has been comparatively less studied than other phases of the implementation process. This may be because of methodological difficulties, funding cycles, and minimal attention to theories and measurement of sustainment. This review synthesizes the literature on sustainment measures, evaluates the qualities of each measure, and highlights the strengths and gaps in existing sustainment measures. Results of the review will inform recommendations for the development of a pragmatic, valid, and reliable measure of sustainment.

**Methods:**

A narrative review of published sustainment outcome and sustainability measures (i.e., factors that influence sustainment) was conducted, including appraising measures in the Society of Implementation Research Collaboration (SIRC) instrument review project (IRP) and the Dissemination and Implementation Grid-Enabled Measures database initiative (GEM-D&I). The narrative review used a snowballing strategy by searching the reference sections of literature reviews and definitions of sustainability and sustainment. Measures used frequently and judged to be comprehensive and/or validated by a team of implementation scientists were extracted for analysis.

**Results:**

Eleven measures were evaluated. Three of the included measures were found in the SIRC-IRP, three in the GEM-D&I database, (one measure was in both databases) and six were identified in our additional searches. Thirteen constructs relating to sustainment were coded from selected measures. Measures covered a range of determinants for sustainment (i.e., construct of sustainability) as well as constructs of sustainment as an outcome. Strengths of the measures included, development by expert panels knowledgeable about particular interventions, fields or contexts, and utility in specific scenarios. A number of limitations were found in the measures analyzed including inadequate assessment of psychometric characteristics, being overly intervention or context specific, being lengthy and/or complex, and focusing on outer context factors.

**Conclusion:**

There is a lack of pragmatic and psychometrically sound measures of sustainment that can be completed by implementation stakeholders within inner context settings (e.g., frontline providers, supervisors).

Contribution to the literature

- The study reviews and summarizes existing sustainment measures including their length, psychometric properties, unit of measurement, and respondent knowledge required to complete the measure, contextual level, and other considerations.
- The study provides a comparison of the constructs within and between sustainment measures.
- The analysis identifies the need for a pragmatic, reliable, and valid measure of sustainment that advances theory and may be completed by frontline providers.

## Background

The implementation of innovations and evidence-based practices (EBPs) [interventions] is generally a long and complex process. Multiple phases have been identified and used to characterize complex implementation processes [1–6]. A common thread or goal amongst the derivations is a culminating phase wherein the intervention is “sustained” and integrated as routine practice. This final phase has been included as part of the implementation process and as an implementation outcome. For example, the Exploration, Preparation, Implementation, Sustainment (EPIS) framework identifies four phases with sustainment being the final phase [7].

Like many other dissemination and implementation (D&I) concepts, the range of terminology related to sustainment and sustainability has challenged the field. The most frequent terms include sustain, sustainment or sustainability, maintain or maintenance, continue or continued, and long-term or follow-up implementation [8, 9]. However, a multitude of other terms have also been used including adhere [9], penetration [10, 11], niche saturation [11], institutionalization [12], routinization [13], normalization [14], integration [15, 16], and community ownership [17]. These terms largely relate to the integration of an intervention into routine practice, however, their operationalization differs. For example, within Proctor’s implementation outcome taxonomy, penetration is defined as “the integration of a practice with a service setting and its subsystems” [10], which similarly aligns with the concept of niche saturation and institutionalization. However, the number of recipients of the intervention (similar to RE-AIM concept of reach) [18] or the providers delivering/using the intervention (similar to RE-AIM concept of adoption) [18] are used as measures of penetration.

Similarly, a number of definitions [8, 9, 19–22] and frameworks [13, 23–25] of sustainment exist. The common and key definitions [8, 9, 24–30] have been methodically developed and there is growing consensus and application of these conceptualizations. Of note, the definition by Moore et al. “after a defined period of time, a program, clinical intervention, and/or implementation strategies continue to be delivered and/or individual behavior change (i.e., clinician, patient) is maintained; the program and individual behavior change may evolve or adapt while continuing to produce benefits for individuals/systems” [21]. A further important definition along these lines is the definition by Shelton et al., “Sustainability has been defined as the continued use of program components at sufficient intensity for the sustained achievement of desirable program goals and population outcomes” [27].

Sustainment and sustainability definitions within the measurement literature are more disparate. The Society for Implementation Research Collaboration (SIRC) instrument review project (IRP) defines sustainability as “the extent to which a newly implemented treatment is maintained or institutionalized within a service setting’s ongoing, stable operations” [31]. The Dissemination and Implementation Grid-Enabled Measures database initiative (GEM-D&I) adds to this definition of sustainability with, “the existence of structures and processes which allow a program to leverage resources to most effectively implement evidence-based policies and activities over time” [32]. Other distinct definitions include: “sustainability as a process or ongoing stage, ” [33] “the extent to which an evidence-based intervention can deliver its intended benefits over an extended period of time after external support from the donor agency is terminated,” [11] “maintenance of treatment goals after discharge,” [20] “sustainability of those intervention outcomes or long-term effects,” [34] and “a suggested ‘adaptation phase’ that integrates and institutionalizes interventions within local organizational and cultural contexts” [25].

As indicated by the multiple review papers on sustainability and recent reconceptualization of sustainment in frameworks [8, 21, 25, 27, 35], there has been a general shift away from thinking of sustainment as an “end game” and more static conceptualizations of sustainment. Sustainment is a dynamic outcome that changes over time to meet the needs of changing context, needs, and evidence. Adaptation is central in the operationalization of sustainment. In light of this shift, there has also been discussion of the movement away from thinking about sustainability as synonymous with “institutionalization,” but that integration into routine practice must occur alongside adaptation to increase fit and to allow for continuous improvement.

There has also been debate on the distinction between sustainment and sustainability. One recent paper describes sustainment as “sustained use,” while sustainability as “sustained benefits” [36]. Another commonly used description highlighting the distinction was developed by Chambers [25] who refers to sustainability as the “characteristics of the intervention that may lend itself to be used over time” as compared to sustainment which is referred to as an “outcome—was the intervention sustained over time,” further saying that “sustainment is an outcome of a sustainability effort” [37]. This is an important concept whereby the process of planning for sustainment should occur throughout an implementation effort through considering the influences of sustainment, deemed sustainability. Palinkas and colleagues [38] also sought to conceptualize the concept of sustainability to encourage further examination and measurement within implementation efforts. They emphasize the elements of continuity and funding as well as conceptualizing sustainability as a process with determinants and an outcome (i.e., sustainment). While several common elements of sustainability determinants and outcomes emerged (e.g., including infrastructure, community buy-in, funding), they found that what distinguishes a determinant from an outcome varies, where outcomes are often distinguished as those elements that continued once funding and/or available support ended.

For the purpose of this review, we conceptualize sustainment as an outcome indicating that the intervention was continued over time. We summarize the specific components of sustainment based on our conceptualization and synthesis of the literature as being: (1) the input(s) (e.g., intervention, program, implementation strategy) continue to be delivered, through (2) routinization and institutionalization of the input(s), while adapting and evolving, and there being (3) ongoing capacity across levels (e.g., organizational, community and systems change) to support the delivery, so that (4) the outputs on the part of the health provider and/or health consumer (e.g., individual behavioral change, clinical benefits, value, impact) are maintained.

Sustainment has been comparatively less studied than other phases of the implementation process [39]. There are multiple explanations for the paucity of work on this topic [8, 24, 27, 28]. Firstly, the time-limited nature or parameters of research grant funding cycles greatly impedes the ability to examine sustainment, especially the effects of long-term sustainment. The nature of current funding mechanisms are such that the time and funding allocated often necessitate a primary focus on one implementation phase, most frequently implementation, and do not allow for complete assessment of the degree to which newly implemented practices are sustained in a way that leads to positive outcomes for patients [40]. As such, sustainment is often not considered or is deemed beyond the scope of a given implementation project. Further, those projects with a primary or secondary focus on sustainment are often limited to short-term examination (e.g., a few months to 1 or 2 years following implementation) or are separate projects and not linked to the prior implementation processes.

A second issue with investigating sustainment is methodological difficulties. Traditional study designs typically focus on more proximal or time-limited implementation outcomes such as fidelity, reach, or engagement [10]. Some designs are inherently limited in the length of prospective follow-up. Designs such as interrupted time-series, and roll-out designs may provide the opportunity to examine sustainment more efficiently than some other more common randomized designs [41]. It is important that these alternative study designs consider sustainment as the outcome that matters most in regard to improving public health impact, for return-on-investment, and in improving efficiency and effectiveness of implementation efforts [42]. Methodological difficulties also arise because planning for sustainment is difficult due to the unpredictability of contextual changes (e.g., new or amended legislation, leadership turnover) or new learning that may occur.

Third, despite multiple reviews of the concept of sustainment, measurement of sustainment has received minimal attention and there appears to be a lack of psychometrically sound and pragmatic sustainment measures [43]. Unlike other phases across the implementation spectrum, sustainment does not have a well-defined time period (i.e., the sustainment period can last indefinitely). Related, sustainment is ecologically complex, thus posing challenges to longitudinal measurement. Because the research examining intervention sustainment is still accumulating, it is not yet known what critical factors at each ecological level are essential to capture the dynamic process of sustainment.

To this end, this review synthesizes the literature on intervention sustainment measures and evaluates the qualities of each measure. We review and extend the current work on sustainment definitions, frameworks, and measures. This work highlights the strengths and gaps in existing sustainment measures to inform recommendations for the development of pragmatic, valid, and reliable measures of sustainment.

## Methods

Known repositories of implementation measures, the Society of Implementation Research Collaboration (SIRC) instrument review project (IRP) [31, 43] and the Dissemination and Implementation Grid-Enabled Measures database initiative (GEM-D&I) [44, 45] were searched for measures of sustainment (or similar terms e.g., sustainability, maintenance). The SIRC-IRP aims to advance implementation science through measure development and evaluation. The IRP centers on the implementation outcomes framework put forth by Proctor and colleagues [10] and constructs outlined in the Consolidated Framework for Implementation Research (CFIR; Damschroder et al. [46]). The GEM-D&I is a project co-developed and introduced by the Cancer Research Network Cancer Communication Research Center at Kaiser Permanente Colorado and the National Cancer Institute’s (NCI) Division of Cancer Control & Population Sciences. The GEM-D&I database looks to create a growing and evolving community of users and resource to share standardized D&I measures that can lead to comparable datasets and facilitate collaboration and comparison across disciplines, projects, content areas, and regions. GEMS-D&I currently has 132 measures inventoried by construct. For this review, we looked at measures inventoried as sustainability.

In addition, we conducted a narrative review of studies purporting to measure sustainment to add to our catalogue of existing measures. Reference sections of literature reviews and definitions of sustainment and sustainability were screened and measures judged to be used frequently, comprehensive, and/or validated were extracted for analysis.

Data extracted from the measures included a description of the measure, the measure’s development process, respondent type(s), psychometric properties, timeframe examined, number of items and factors, scoring, and considerations. The generalizability of the measure and any other limitations or concerns were considered. For the measures that were quantitative scales, as opposed to open-ended questions or telephone surveys and that included ongoing delivery/use of an EBP and other dimensions of sustainment as an outcome, we also extracted the constructs of the scales. Extraction was performed inductively by two team members (JCM and AG). The extracted constructs were then collated under the components of our sustainment definition. Extraction continued until thematic saturation was reached and results were confirmed by a third team member (KSD). Team members and authors included national and international implementation scientists, including several with expertise in pragmatic measurement and measure development.

## Results

Eleven measures targeting or that included sustainment were identified across the GEM-D&I, SIRC-IRP, and other sources as described above. Three of the measures were found in the GEM-D&I database, three in the SIRC-IRP (one measure was in both databases), and six came from other sources. We briefly describe each of the measures and summarize them in Table 1.

**Table 1:** Summary of sustainment measures

Name of measure/primary publication	Description of sustainment measure or sub-measure	Construct measuring	Setting/context	Measure development	Respondent type	Psychometric properties	Number of items	How is the measure scored/rated?	Considerations
RE-AIM Framework Dimension Items Checklist - Maintenance Measure [18, 35, 47] (http://www.re-aim.org/resources-and-tools/measures-and-checklists/)	To plan for and evaluate **maintenance** of a health program or policy at the individual and setting level. Maintenance is described as the extent to which a program is institutionalized or becomes a routine part of practice (setting level), and extent to which individuals see long-term effects of the program 6+ months after most recent contact (individual level).	Maintenance = sustainment	Public health	Developed by The Implementation Science Team at NCI Division of Cancer Control and Population Sciences with key RE-AIM leaders and authors as part of project to review grant proposals using RE-AIM	Public health and health services researchers. The website also states that RE-AIM is intended to be considered by “program planners, evaluators, readers of journal articles, funders and policy-makers.”	N/A. However, there has been a systematic review of the RE-AIM Framework (Gaglio, Shoup and Glasgow, 2013).	Individual: 5 Setting: 4	Qualitative (open-text responses) in the RE-AIM Planning Tool and quantitative (dichotomous, yes/no) in the Measuring the Use of the RE-AIM Model Dimension Items Checklist.	Checklist developed as a guide rather than a specific measure. Not a direct\replicable outcome measure of sustainment. However, the checklist or planning tool could be used as an outcome measure that is tracked over time by researchers or implementers.
Level of Institutionalization Scale (LoIn) [12] (Goodman, McLeroy, Steckler, and Hoyle, 1993)	Measures **institutionalization** or the extent to which an innovative program has gone through the stages of passages, routines, and niche saturation to become an integral part of organization.	Institutionalization = sustainment	Health programs	Initial questionnaire reviewed by 5 multidisciplinary experts, revised and pilot tested in local health department	Administrators in health programs	8 subscales. 4 routine subscales (average interfactor correlations = .85) 4 niche saturation subscales (average interfactor correlations = .76). Chronbach’s alpha moderate to high (not listed). Internal consistency and factor structure.	45 items (15 3-part items)	4-point Likert-style response scale	Complex measure to complete, respondent burden. Can only be completed by higher-level administrator
Evidence-based Practice (EBP) Sustaining Telephone Survey [48] (Swain, Whitley, McHugo, and Drake, 2010)	Mixed methods telephone survey to assess **sustainability** of EBPs in mental health agencies. Has sections on sustainment of practice, adaptation of practice, and facilitators of sustainment. Administered two years after implementation.	Sustainability = sustainment and sustainability	Mental health agencies	Not reported	Program leaders, administrator, and trainers	Not reported	47 items, quantitative and qualitative	Qualitative answers transcribed and coded, quantitative answers analyzed with descriptive statistics. Sustainment factors measured with 5-point Likert-style response scale.	Long, difficult to score, telephone survey (retrospective self-report).
The Team Check-Up Tool (TCT) [49] (Lubomski et al., 2008)	Designed to be completed over the course of a team-based **quality improvement** (QI) intervention to assess dynamic context and progress.	Dynamic context and progress = sustainability	Healthcare QI teams	Developed by a statewide QI collaborative. Items were developed by clinicians and health service researchers, and later modified and refined in response to feedback and implementation experiences. Experts rated items on 4-point Likert scale, and team members participated in focus groups to provide item feedback.	QI team members	Evidence supporting the temporal stability, construct validity, and responsiveness of TCT Content Validity Index = .87. Positive qualitative user feedback.	15 topics with subscales, 36 total items assessed for validity	Experts rated on 4-point Likert-style response scale	Looks at individual components of the specific intervention in the study, how many people are doing them, and what the barriers are. Appears to be closer to a measure of fidelity and barriers to rather than sustainment.
Stages of Implementation Completion (SIC) [50] (Chamberlain, Brown, and Saldana, 2011)	Purpose: To document implementation progress of an EBP within an implementation project. The SIC measures 31 activities across 8 stages of implementation that span three phases: pre-implementation, implementation, and sustainability. **Sustainability:** defined as competency and credentialing as a sustainable program.	Sustainability = sustainment	Randomized control trials testing EBP implementation in health settings	SIC developed by members of study team and California Institute of Mental Health with 12 stages. Applied SIC to sites; iterative process using initial observations lead to 8 stages. Used in the context of a study comparing effectiveness of 2 implementation strategies for MTFC. Constructed to reflect the same stages for both strategies: identical requirements for stage completion, equivalent activities within the stages, etc.	Information is collected from individuals at multiple levels (providers, agency leaders, etc.). As an example, the SIC was completed by 53 sites from 51 counties in California and Ohio that implemented Multidimensional treatment foster care [REF].	Difficult to use traditional psychometric models because many SIC proportion items are dichotomous and SIC duration items are time-distributed. Reliability and validity were estimated using IRT-based Rasch models; adequate reliability.	8 stages, 31 activities across all stages	Three scores are calculated: 1) speed of implementation (duration of each stage), [2] proportion of implementation activities completed within each stage and [3] the number of stages (out of 8) completed.	Does not include all implementation activities or provide information on why activities were not completed. It also requires fee-for-service consultation/contracting with the measure developers to track and complete the SIC.
NHS Sustainability Model and Guide [51] (Maher, Gustafson, and Evans, 2010; https://improvement.nhs.uk/resources/Sustainability-model-and-guide/)	Diagnostic tool to predict the likelihood of **sustainability** of an improvement initiative across three factors, process, organization and staff.	Sustainability= sustainment and sustainability	Health organizations	Co-produced for the NHS by front line teams, improvement experts, senior administrators, clinical leaders, people with content expertise from academia and other industries, as well as the literature on change and sustainability.	Individuals or teams working in NHS/healthcare	Not reported	10 subscales, 4 questions each (40 items)	Each factor has four detailed response scenarios. Participants mark the response that corresponds to their project. Each response is given a specific score. These are added to give a final sustainability score.	Developed as a formative self-assessment and guide for implementers rather than a research tool. No psychometric properties. Blends predictors and sustainment indicators.
A framework and a measurement instrument for sustainability of work practices in long-term care [15] (Slaghuis, Strating, Bal, and Nieboer, 2011)	Measure of **sustainability** of new work practices in long-term health care practices where sustainability is the dynamic process through which actors develop/adapt organizational routines to a new work method. Two domains, **routinization:** new work method becomes part of everyday activities; subscales of principles, practices, and feedback; and **institutionalization:** new work method is embedded in the organizational context, structures, and processes; subscales of skills, documentation materials, practical materials, and team reflection	Sustainability (routinization and institutionalization) = sustainment and sustainability	Healthcare organizations	Literature review conducted by authors on sustainability and related themes in health care. Created statements to be evaluated on five point Likert scale; Content validity assessed by authors and 11 experts, majority had worked in long-term care organizations as care professionals, quality staff or management. About half of the experts also had practical professional experience in organizing quality improvement projects.	Staff at long-term care organizations (e.g., nursing homes, elderly homes, care for disabled) and members of improvement teams	For 52 items tested: Hierarchical 2 factor model w/ routinization and institutionalization as separate constructs = best fit. Average factor loadings of each item = .54. Structure coefficients ranged from .68-1. Short version: bivariate correlations between routinization and institutionalization = .79. Scores within subscales of these ranged from .49-.80.	Tested: 52 Long version: 40 Short version: 30 Translation: N/A	Each sub-dimension had a scale of 5-10 statements describing several aspects. Evaluated on a five point Likert scale, ranging from '1: I don't agree at all' to '5: I agree very much', including the option 'I don't know'.	Low response rate (33%) and small sample size (*n* = 112, across 63 teams, with missing data); need to assess test-retest reliability. Some subscales not generalizable across contexts and innovations.
Normalization Process Theory-based Measurement (NoMAD) [14, 72] (Finch et al., 2013)	Measures intervention normalization using **Normalization Process Theory (NPT)** constructs. NPT states that practices become implemented, embedded, and integrated when people work individually and collectively to enact them. Four generative processes: coherence, cognitive participation, collective action, and reflexive monitoring.	Normalization = sustainment and sustainability	Healthcare organizations	Developed items to reflect each of 4 NPT constructs. Process: lit review, identifying concepts, generating and appraising items, iterative item testing	6 sites implementing health-related interventions; respondents had a variety of roles (clinical, managerial, etc.)	Good face validity, construct validity, and internal consistency. Construct validity for 4 constructs was supported. Chronbach’s alpha for 4 NPT construct groupings ranged from .65-.81. Overall normalization scale high reliability (20 items, alpha = 0.89) Bivariate correlations between NPT construct measures ranged from .49 to .68. Correlation between 43 NPT and 3 normalization items was low.	Tested: 46 Final: 23	5-point Likert-style response scale of agreement (strongly agree - strongly disagree) for NPT construct items 11-point Likert-style response scale for normalization assessment items	Needs to be validated across implementation settings; need to assess test-retest reliability
Program Sustainability Assessment Tool (PSAT) [52] (Luke, Calhoun, Robichaux, Ellito, and Moreland-Russell, 2014)	Assesses the capacity for **sustainability** of public health-related programs.	Sustainability = sustainment and sustainability	Public health programs	Developed pilot based on lit review and concept mapping	Tested in large number of community and state-level programs. 592 program managers and staff in 252 public health programs aimed at addressing tobacco control, diabetes, obesity prevention, or oral health	Good fit of the 8 domain model. Sig diff in item-factor loadings across state and community levels (*p* < .001). Average Chronbach’s alpha of subscales = .88 (range 0.79-0.92). CFI for final 40 items = .89.	Pilot: 63 items, 9 sustainability domains Final: 40 items, 8 sustainability domains (5 items/domain)	Items rated on 7-point Likert-style response scale of agreement	Relatively long scale. Can only be completed by higher-level administrators.
Clinical Sustainability Assessment Tool (CSAT) [53] (http://www.sustaintool.org/csat/)	Assesses the capacity for **sustainability** of clinical programs	Sustainability	Clinical programs	Developed based on lit review and concept mapping	Pilot tested with 120 individuals (physicians, pharmacists, nurses, administrative staff, leadership, and others) assessing clinical practice from a range of clinical settings and environments.	Developers are in the process of validating the tool.	Pilot: 35 items, 7 domains (5 items/domain)	Items rated on 7-point Likert-style response scale ranging from no extent to a full extent.	Relatively long scale.
Program Sustainability Index (PSI) [54] (Mancini and Marek, 2004)	Purpose: measure **sustainability** of community-based programs using the following conceptual, model of 3 linked domains: **1. Sustainability:** “capacity of programs to continuously respond to community issues… maintains a focus consonant with original goals and objectives… key element… is providing continued benefits” focus on sustained benefits to families vs. sustained activities. 2. **Middle-range program results**. Impacted by sustainability. 3. **Ultimate results**. Impacted by first two domains; pertains to program sustainability**.**	Sustainability= sustainment and sustainability	Community-based programs	Sustainability elements determined in previous mixed methods studies w/ 100+ interviews of community program personnel and 4000 responses to open-ended questions. Pilot survey developed with qualitative results then administered across 153 community-based programs ➔ 7 elements consistently influencing sustainment.	243 human development and family life professionals at varying levels of program development/evaluation voluntarily completed survey. Data collected at annual Children, Youth and Families at Risk initiative meeting	Initial analyses: unsatisfactory fit. Factor analysis suggested 6 domains. Acceptable internal consistencies (0.67-0.88), acceptable performance validity for the 29 items.	Tested: 53 items, 7 domains Final: 29 items, 6 domains	3-point Likert-style response scale of extent. Items within each of the six PSI subscales were averaged to create scores.	Respondents fairly homogenous, should be tested in other settings. Frequently adapted or certain subscales selected, indicating lack of utility or fit in current form

Note = denotes the corresponding construct according to the definitions in this work where sustainment is an outcome, while sustainability is the characteristics of the intervention that lend itself to be sustained.

GEM-D&I had six measures indexed as targeting sustainability, and three of these met criteria to be included in our review. The included measures are as follows: The Reach Effectiveness Adoption Implementation Maintenance (RE-AIM) framework [18, 35, 47], Level of Institutionalization Scale (LoIn) [12], Program Sustainability Assessment Tool [52]. Three other measures linked to sustainability in the overall GEM database were excluded, with reasons as follows: The COMMIT questionnaire [55], as it is specific for the COMMIT study; the Hospital Elder Life Program Sustainability Measure [56], as it was a qualitative interview guide rather than quantitative measurement tool; and the Community-Based Participatory Research (CBPR) Model-Sustainability Measure [57], as it prospectively measures likelihood for sustainability.

The SIRC-IRP had thirteen measures rated as measuring sustainability. Three measures were included in our review: Evidence Based Practice Sustaining Telephone Survey [48], Program Sustainability Assessment Tool [52], and Program Sustainability Index [54]. The remaining ten were excluded: School-wide Universal Behavior Sustainability Index-School Teams [58] and the Amodeo Counselor Maintenance Measure [59], as they were specific for a single intervention; the Change Process Capability Questionnaire [60], as it is a measure of an organization’s capability for successful implementation rather than an outcome measure; Eisen Provider Knowledge and Attitudes Survey [61], as it measures prospective intentions; Knowledge Exchange Outcomes Tool [62], as it does not measure constructs of sustainment; the Organization Checklist [63], as it is unpublished; the Prevention Program Assessment [Maintenance Scales] [64] and the Sustainability and Spread Activities Questionnaire [65], as items were not available for review; the Sustainment Cost Survey [66], as it measures costs only; and the General Organizational checklist [67], as it measures quality of delivery.

In addition to measures from the two databases, six measures were included from our narrative review: The Team Check-Up Tool (TCT) [49], Stages of Implementation Completion (SIC) [50], NHS Sustainability Model and Guide [51], a framework and a measurement instrument for sustainability of work practices in long-term care, NoMAD [14, 72], and the Clinical Sustainability Assessment Tool (CSAT) [53] (http://www.sustaintool.org/csat/). See Table 1 for a summary of included sustainment measures.

### Part 1: Review of individual sustainment and sustainability measures (*n* = 11)

#### RE-AIM (Reach Effectiveness Adoption Implementation Maintenance) Framework and Tools [18, 35, 47]

RE-AIM was originally developed as a framework that consists of five elements or dimensions (reach, effectiveness, adoption, implementation, maintenance) that relate to behavioral health interventions or programs. Since the original publication of RE-AIM in 1999 [18], a number of tools have been developed to support the application of the RE-AIM framework in research-to-practice projects of health promotion and disease management interventions. Two of the resultant tools that have been developed are the RE-AIM Planning Tool and the Measuring the Use of the RE-AIM Model Dimension Items Checklist. In both tools, there is a separate section that includes specific questions for planners or evaluators related to the “maintenance” element of RE-AIM. In RE-AIM, maintenance is defined as the extent to which an intervention, program or policy becomes routine practice or policy at the setting and/or individual level, which corresponds to sustainment. The RE-AIM Planning Tool consists of 9 items (4 directed towards individual-level maintenance and 5 directed towards setting-level maintenance) that are primarily open-ended questions (e.g., “What do you plan to do to support initial success and prevent or detail with relapse of participants;” “What do you see as the greatest challenges to the organizations continuing their support of the program?”). The RE-AIM Model Dimension Items Checklist also consists of 9 items (5 directed towards individual-level maintenance and 4 directed towards setting-level maintenance) that are treated as a checklist for evaluation purposes (e.g., “Use of qualitative methods data to understand long-term effects;” “Some measure/discussion of alignment to organization mission or sustainability of business model”). These items refer to the maintenance time period as those activities that occurred following 6-month follow-up. There are no reported psychometric properties on the planning or evaluation tools. However, a recent systematic review characterizes the use of the RE-AIM framework as a whole [68]. More recently, Shelton, Chambers, and Glasgow [35] published a perspective that calls for an expanded conceptualization of the RE-AIM framework, with explicit focus on equity and cost of implementation, to enhance the study of maintenance/sustainability. The authors recommendations of RE-AIM were as follows: to extend the time course of maintenance measurement, to at least 1 year post initial implementation and over time, to capture the dynamic “evolvability” of the EBP and implementation strategies; iterative and periodic re-assessment of RE-AIM dimensions to inform and document adaptations within a changing multilevel context; and explicit focus on equity and costs across RE-AIM dimensions (e.g., representativeness of patient/population subgroups) to promote long-term sustainability.

#### Goodman’s Level of Institutionalization (LoIn) [12]

The LoIn scale measures the extent to which an innovative health promotion program is integrated within its host organization. It is based on the theory that organizations are composed of production, maintenance, supportive, and managerial subsystems and that institutionalization occurs when a program becomes embedded into these subsystems. The level of institutionalization measures the extent to which an innovative program has gone through the stages of passages, routines, and niche saturation to become an integral part of an organization. Institutionalization corresponds to both sustainability and sustainment. The measure was developed and finalized following expert review and subsequent pilot testing. The resulting measure includes 45 items with eight subscales targeting routines and niche saturation across the subsystems. Routines and niche saturation items are scored on 4-point Likert-type scales. In terms of time period, the measure also includes sub-items related to the number of years activities have persisted. Psychometric properties were assessed through an examination of Cronbach’s alpha, interfactor correlations, and the relationship of factors to program years and perceived permanence. While LoIn developers describe internal consistency as “moderate to high,” Cronbach’s alpha ranged from .44 to .86. Furthermore, LoIn developers acknowledge that the scale is complex to complete and can only be completed by higher-level administrators.

#### EBP Sustaining Telephone Survey [48]

This measure is a 47-item interview that includes qualitative and quantitative items to report on the sustainability of the practices within the National Implementing Evidence-Based Practices Project for people with serious mental illness. The measure is divided into three sections assessing [1] whether or not the site continued the practice (and reasons for sustaining or not sustaining), [2] whether or not the practice had been modified to suit local contexts, and [3] factors affecting sustainability. The timeframe for using the measure is 2 years post implementation. The psychometric functioning of this measure has yet to be established, and scoring participant responses may be challenging particularly with regard to triangulation of quantitative and qualitative data.

#### Team Check-up Tool (TCT) [49]

Lubomski et al. developed the Team Check-up Tool (TCT) for monitoring team progress within a quality improvement (QI) intervention. Although the tool was developed for intensive care unit (ICU) patient safety collaborative teams, developers intended for it to be applicable across clinical areas. The original TCT is a two-part tool that elicits information about QI activities required for training, resources, or other aids to facilitate sustainment. The first part of the tool contains nine questions that ask respondents about specific facilitators, in terms of administrative activities and senior executive actions related to the QI intervention. The second part of the tool provides a list of ten potential barriers that slowed team progress in the previous month. The TCT aims to target factors that are predictive of the team’s failure or success. The TCT was later modified and the psychometric functioning of this modified version was examined by Chan et al. [69]. The modified tool was expanded to contain 14 items in part 1 that assess education activities, use of the intervention, plus the original items assessing QI administration and senior executive activities. Part 2 of the modified TCT contains 13 potential barriers, and a part 3 was added, containing five items on contributors to poor team function. Cronbach’s α was .91 for team barriers. Spearman correlation assessing temporal stability was between .39 and .92 with 10 of the 13 items demonstrating at least moderate correlation between months. Change in barriers over time was not statistically significant, perhaps indicating a lack of measure responsiveness. Convergent and discriminant validity were supported, though predictive validity was not. Content validity, feasibility, and importance of the modified TCT was later assessed by Marsteller et al. [70] via two focus groups and a feedback session. Content validity index was found to be .87, though several barriers to completing the tool were uncovered and included the length of the tool, the month-to-month redundancy, the lack of feedback, and confusion about the tool’s questions.

#### Stages of Implementation Completion (SIC) [50]

The SIC is an observational, data-driven measure of implementation progress that is designed to be completed by an implementation team. It encompasses eight stages and 31 activities that span three implementation periods (pre-implementation, implementation, sustainability). The SIC yields three scores: (1) speed of implementation (duration of each stage), (2)] proportion of implementation activities completed within each stage, and (3) the number of stages (out of 8) completed. The SIC corresponds to the construct of sustainment. The time period examined pertains to the duration of each stage. The SIC developers recommend that researchers or program implementers contract with the SIC team for measure customization, data collection, and analysis. Data are collected on an ongoing basis during implementation from a variety of stakeholders including organizational and system leaders and providers. Completing the SIC can be a lengthy, iterative process. The SIC was evaluated using Rasch analysis, using a study of the implementation of Multidimensional Treatment Foster Care (MTFC). The data resulting from the measure (e.g., dichotomous proportion items of whether activities were completed or not and time-distribution duration items) do not easily fit psychometric measures of classical test theory. The initial analysis of the MTFC-SIC measure indicated preliminary evidence of reliability and validity.

#### National Health Service (NHS) Sustainability Model and Guide [51]

The NHS Sustainability Model is a self-assessment “diagnostic tool” to identify strengths and weaknesses in an implementation plan, to predict the likelihood of the initiative’s sustainability corresponding to the construct of sustainability. The Sustainability Guide is intended to provide practice advice on increasing the likelihood of an initiative’s sustainability. The Sustainability Model and Guide each consist of the 10 factors relating to process, staff, and organizational issues that play a role in sustaining change in healthcare. The Model and Guide were co-produced for the NHS by frontline teams, improvement experts, senior administrators, clinical leaders, people with content expertise from academia and other industries, as well as the literature on change and sustainability. In terms of the timeframe, the developers recommend repeated use of the tool for formative evaluation of quality improvement initiatives. Participants rate the level of each factor, by marking the description of each factor that best describes their local project. No specific time period for using the measure is referenced or assessed. A scoring system is provided to calculate scores for each factor, which are then added together to arrive at an overall sustainability score. Participants are encouraged to plot their scores to identify factors with greatest potential for improvement. The psychometric functioning of the tool has not been established; however, they state that a score of 55 or higher offers reason for optimism.

#### A framework and a measurement instrument for sustainability of work practices in long-term care [15]

Slaghuis et al. [15] developed a measure to test the integration of small improvement interventions implemented via the Breakthrough method in long-term care organizations in the Netherlands. The sustainability framework is based on the concept of work practices and organizational routines where sustainability is seen as “a dynamic process in which actors in a target work practice develop and/or adapt the organizational routine to a new work method”. This process is centered on the concept of routinization and institutionalization [12, 71]. Routinization is the molding of the new work practice to fit the organization, while institutionalization is the embedding of the new practice in the organization, incorporating the support and context aspects required for sustainment. Three domains were attributed to routinization (routinization via principles, practices, and feedback) and four to institutionalization (institutionalization of skills, documentation materials, practical materials, and team reflection). These domains correspond to the construct of sustainment. For each domain measurement, developers designed a scale of 5–10 statements rated on a 5-point Likert response scale, with no specific time period for assessment mentioned or referenced. Structural and content validity were assessed by the developers and 11 experts. The psychometric properties of an initial 52-item instrument were assessed in a sample of 112 individuals, from 63 teams, with imputed missing data. Construct validity was assessed with structural equation modeling (SEM) and principal component analyses (a hierarchical two-factor model with routinization and institutionalization as separate constructs was best fit with high factor loadings, but some cross-loadings), and reliability with Cronbach’s alpha (all subscales were above 0.7). A short version (31 items) and long version (40 items) are available. Further psychometric analyses would be beneficial to address the low response rate (33%) and small sample size (*n* = 112, across 63 teams, with missing data). A limitation of this measure is that items are not generalizable across all contexts and innovations.

#### NoMAD [14, 72]

Finch et al. developed and validated the NoMAD measure to assess activity related to the normalization of complex interventions based on the Normalization Process Theory (NPT), corresponding to the constructs of sustainability and sustainment. The NoMAD developers intend for the NoMAD to be a pragmatic measure of implementation processes and encourage users to apply it flexibly to their implementation research and practice needs. The NoMAD developers describe the NoMAD “as an adaptable ‘bank of items’” [72]. Data from six implementation projects were assessed to determine the items and factor structure, as well as to examine the psychometric functioning of the resulting NoMAD measure. These six implementation projects varied with regard to the kinds of interventions being implemented (digital health, smoking cessation, patient self-management, oral health risk, system-level IT, sports program), implementation timelines, and professionals involved in the implementation activities (clinical, administrative, managerial, and other professionals in non-health contexts). Resulting items consist of 20 construct items and 3 normalization items, though users are encouraged to adapt the text for their own research and practice needs. Each of the 3 normalization items is answered using an 11-point visual analog scale ranging from 0 to 10, and assesses the extent to which the intervention feels familiar, is currently a normal part of work, or will become a normal part of work. The 20 construct items include four sub-sections assessing the four NPT constructs: coherence (4 items; *α* = .71), cognitive participation (4 items; *α* = .81), collection action (7 items; *α* = .78), and reflexive monitoring (4 items; *α* = .65). Individuals are instructed to complete the construct items by selecting an answer from option A or option B depending upon the relevance of the item. Specifically, option A includes a 5-point Likert-type response option ranging from strongly agree to strongly disagree. Option B includes three response options, “not relevant to my role,” “not relevant at this stage,” and “not relevant to the intervention.” An overall normalization scale comprising items from across the four construct sub-sections is also available (20 items; *α* = .89). No specific time period is assessed or referenced. The measure shows promise as a measure of sustainability and potentially sustainment if cut-off scores are defined. Further testing across contexts and test-retest reliability are recommended.

#### Program Sustainability Assessment Tool (PSAT) [52]

The PSAT assesses the capacity for program sustainability of various health programs corresponding to the construct of sustainability. The measure was based on the developers’ prior literature review and concept mapping study that resulted in 40 items across 8 domains, with 5 items per domain, with no specific timeframe. Domains include political support, funding stability, partnerships, organizational capacity, program evaluation, program adaptation, communications, and strategic planning. In the original measure development publication, internal consistency was reportedly high with Cronbach’s alpha values ranging from .79 to .92. Measure developers conducted multiple-group confirmatory factor analyses to test factorial invariance across program level (state and community) and program type (e.g., tobacco, obesity, diabetes, oral health). While no difference in psychometric functioning was found between program types, the PSAT functioned differently between community and state programs. A limitation with the measure is that it targets current capacity for future sustainment as opposed to evaluating the quality of program sustainment following initial implementation. The tool also requires higher-level organizational knowledge so it cannot be completed by frontline workers/service providers.

#### Clinical Sustainability Assessment Tool (CSAT) [53]

The CSAT measure was adapted from the PSAT based upon a literature review and expert-informed concept mapping. The resulting tool is a 35-item self-assessment that clinical staff and stakeholders complete to evaluate the sustainability capacity of a practice. The developers define clinical sustainability as “the ability of an organization to maintain structured clinical care practices over time and to evolve and adapt these practices in response to new information” [53]. While the PSAT intends to assess a wide range of public health programs, the CSAT intends to assess the sustainability capacity of a clinical practice. The CSAT assesses the following seven domains: engaged staff and leadership, engaged stakeholders, organizational readiness, workflow integration, implementation and training, monitoring and evaluation, and outcomes and effectiveness. Respondents rate practices using a 7-point scale indicating the extent to which the practices are supported by these domains of processes and structures hypothesized to increase the likelihood of sustainability. Information regarding the psychometric functioning of the CSAT is not yet available and developers are in the “process of validating the tool” [53].

#### Program Sustainability Index (PSI) [54]

The PSI was developed to assess the attributes of sustainability among community programs. The measure was informed by a model of community-based program sustainability consisting of three cascading and/or sequential and linked domains: program sustainability elements (e.g., leadership, collaboration), middle-range program results (e.g., needs met, effective sustainability planning), and followed by the ultimate result of program sustainability. Development was also informed by the results of mixed methods studies examining elements of community program sustainability. Comprised of seven subscales, the PSI assesses both outer and inner context factors, including leadership competence, effective collaboration, understanding community needs and involvement, program evaluation, strategic funding staff integration, and program responsivity. Two items from the Leadership Competence and Strategic Funding subscales mention a timeframe of at least 2 years for assessment by the PSI. The PSI is administered by qualitative interviews or as a web-based survey to multilevel administrators. It has been used in child welfare, Veterans Affairs medical centers, and hospitals. Informed by factor analytic examination of measure structure and fit, the final measure is comprised of 29 items rated on a 4-point Likert scale. Cronbach’s alpha ranged from .76 to .88, indicating strong internal consistency. Measure limitations include knowledge required of both inner and outer context to complete and the focus is on sustainability versus sustainment.

### Part 2: Sustainment measure constructs

Six measures were quantitative scales measuring sustainment and therefore had their constructs extracted and reviewed. The constructs from the measures were thematically extracted, which inductively resulted in 13 construct domains (see Table 2). In addition to continued delivery, three constructs relate to sustainability processes and benefits of the intervention: (i) monitoring (including fidelity and benefits), (ii) adaptation and improvement, and (iii) reflection and team involvement. Two constructs relate to the integration of the intervention, (i) institutionalization and (ii) routinization, and three constructs relate to the outer context (as defined by EPIS) [5, 7], (i) external support and communication, (ii) partnership, and (iii) financial resources and funding. Outer level constructs were contained in five of the six measures. There were three constructs relating to the inner context (as defined by EPIS [5, 7]), (i) leadership support and organizational climate, whereby there was innovation-fit with the goals and direction of the organization, (ii) organizational capacity in terms of sufficient resources and workforce, and (iii) training provided. Inner context, organizational constructs were contained in all measures. Finally, one construct encompassed the individual-level factors of staff support, their behaviors, and attitudes towards the EBP/innovation. The inner context, individual-level construct was found in five of the six measures reviewed.

**Table 2:** Constructs of sustainment and sustainability measures

	Sustainment definition and constructs												
	The input(s) continue to be delivered	Routinization and institutionalization of the inputs, while adapting and evolving, and the outputs on the part of the health provider and/or health consumer are maintained.					Ongoing capacity across levels to support the delivery of the inputs.						
							Outer context			Inner context			
	Ongoing use/provision	Adaptation and improvement	Monitoring	Reflection and team involvement	Routinization	Institutionalization	External support and communication	Partnership	Financial resources and funding	Leadership support and organizational climate	Organizational capacity	Training	Staff support, behavior, and attitudes
Level of Institutionalization Scale (LoIn) [12]	X	X	X	X		X			X		X		X
NHS Sustainability Model [67]	X	X	X	X	X	X	X			X	X	X	X
Measure of Sustainability of work practices [15]	X	X	X	X	X	X					X	X	
NoMAD [14]	X	X		X	X	X				X	X	X	X
Program Sustainability Assessment Tool [45]	X	X	X		X		X	X	X	X	X		
Program Sustainability Index (PSI) [56]	X	X	X	X	X		X	X	X	X	X	X	X

Note: Construct definitions: *Ongoing use/provision* The core components of the intervention continue to be delivered after a designated period of time, *Adaptation and improvement* The intervention is being adapted to fit the organization and improve the intervention, ideally based on monitoring, *Monitoring* The intervention benefits and intervention process (fidelity) being monitored and evaluated, *Reflection and team involvement* The intervention and its implementation are discussed with team members, *Institutionalization* The intervention is part of the norm of the organization and staff practice, *Routinization* The intervention is integrated into the operations of the organization and staff practice, *External support and communication* Political and/or community support for the intervention, including ongoing communication and demonstration of benefits, *Partnership* Collaboration and connections with community and external stakeholders, *Financial resources and funding* Stability of financial resources or funding, *Leadership support and organizational climate* Intervention fit with organizational goals and culture and leadership support for the intervention, *Organizational capacity* Resources and workforce, *Staff support, attitudes, and behaviors* Staff values, opinions, and actions, *Training* Adequate knowledge and skills, ideally acknowledging the need for ongoing training and training of new staff

## Discussion

To extend the evidence base on pragmatic sustainment measurement, we conducted a multistep narrative review of sustainment measures. Using two large implementation science measure databases (GEM-D&I and the SIRC-IRP) and a supplemental narrative review of the literature, we identified 11 sustainment measures that met our review criteria of being used frequently, assessing sustainability/sustainment, and judged to be comprehensive and/or validated.

The recent acknowledgement of the need for both psychometrically strong and pragmatic measures is highlighted in the results of this review [73]. Out of the eleven measures meeting criteria, three were deemed time-intensive (e.g., many including as many as 40 or more items) and/or complex to complete (e.g., suited for interview style data collection). A related issue was that some measures required stakeholders at multiple levels or knowledge across multiple levels to complete. In general, the items in most measures were not well-suited for frontline providers to complete, but instead, existing measures were best suited for or required high-level administrators or executives with knowledge of EPIS or CFIR outer context factors such as community partnerships, funding arrangements, contracting, or financial resources. The sustainment of interventions, and especially discrete EBPs, relies on the inner context of health organizations and service providers delivering care [74, 75]; however, the available *sustainment* measures primarily assess outer context influences. While critical for implementation, questions related to outer context may be challenging for stakeholders delivering care to answer and can only be completed by a small subset of stakeholders (e.g., government administrators, organizational executives) [8]. Pragmatic measurement of sustainment should include measures from different perspectives, including measures that may be completed by inner context stakeholders such as direct service providers and supervisors whose voice has not been fully represented.

The methodology for developing the reviewed measures varied widely. While some measures were based on sustainability frameworks (e.g., the PSAT was based on the capacity for sustainability framework) [76], others were less explicit about the items’ theoretical origins. Along with the range of sustainability definitions, there is a range of sustainability frameworks (e.g., Dynamic Sustainability Framework [25]) and implementation frameworks that include sustainability (e.g., EPIS [5, 7], CFIR [46], PRISM [77]). Ideally measures of sustainment and sustainability should map onto these frameworks [27]. Furthermore, there are a number of reviews of sustainability influencing factors [8, 27, 78, 79]. It was positive to see our findings, extracted as the sustainment measure constructs (Table 2), broadly align with these reviews. Of note, while some constructs regarding sustainability processes such as monitoring, and adaption and improvement did appear in the reviewed measures, others including planning, technical assistance, and navigating competing demands did not arise. In addition, the structural characteristics of the organization and characteristics of the intervention, implementer(s), and population were not explicitly included.

It is important to consider the time in which an intervention has continued to be delivered/used when measuring sustainment. Results suggest that half of the examined measures did not specify the time period for measuring sustainment. Among the remaining measures, the time period varied from a 6-month follow-up period (the original RE-AIM Framework Dimension Items Checklist) [47] to over 2 years (evidence-based sustaining telephone survey [48] and PSI [54]). For one measure, the SIC [50], the sustainment period was variable and project specific, informed by the time of implementation initiation and completion. Guidance or inclusion of the timeframe of sustainment should be modeled off recommendations that suggest measurement should occur 12 months, but ideally 2 or more years after implementation [8]. However, it is also possible to measure sustainment earlier in the implementation process to capture its dynamic nature and as a formative tool to tailor implementation strategies and plan for sustainment. This is aligned with the extension of the RE-AIM framework’s focus on “evolvability” across the life cycle of implementation with a goal of contributing a sustainable and equitable health impact rather than on sustainment alone as a set end point [35].

While the majority of measures were validated questionnaires, one measure from the GEM-D&I—the RE-AIM Maintenance Measure—has an open-ended structure to examine the individual and organizational-level long-term effects of a program on outcomes. Combined use of a more qualitatively oriented measure along with a brief, validated questionnaire may be a method to consider for research or practice-based projects with greater resources. Further, of the 11 measures identified, there were no measures deemed to be applicable across settings and that can be tailored for particular EBPs. This is greatly needed because of the growing number of implementation efforts involving the implementation of multiple EBPs concurrently or sequentially [74, 80, 81]. While continued delivery and adaptations over time may require an intervention-specific sustainment measure, we feel a generic sustainment measure that captures the broad constructs of sustainment would assist in creating generalizable evidence within implementation science. Sustainment measure development may be aided by the timely questions posed in the extension of the RE-AIM framework [35] that focus on health equity and dynamic context (e.g., Are the determinants of sustainment the same across low-resource and high-resource settings?).

There are challenges in rating the pragmatic qualities of implementation science measures. We applied the guidance from the recommended criteria for pragmatic measures by Glasgow and Riley [73] and the Psychometric And Pragmatic Evidence Scale (PAPERS) [82]. However, we found that the former was better-suited for evaluating patient/client-directed measures while the latter required data that was not available for us to complete ratings (e.g., level and types of stakeholder involvement in measure development).

The review highlighted that there appears to be currently no pragmatic, psychometrically strong measure of sustainment that can be easily completed by inner context providers. For good reason, the reviewed measures contain both inner and outer context factors. The majority of the measures require knowledge of external or bridging factors such as communication and partnerships with community members and politicians, and securing funding. The requirement of multilevel knowledge creates issues for respondents and we feel separate scales completed by stakeholders at different levels within the context have a place within implementation science. The review of the constructs provides guidance on the key constructs for inclusion in a pragmatic inner context measure of sustainment (Table 2). In summary, we believe a measure of inner context sustainment provides an important perspective in measuring sustainment. Such a measure could be used in combination with intervention-specific assessment of core component continuation (e.g., sustained fidelity) and adaptation, measures of intervention outcomes (e.g., patient or provider benefits), and measures of outer context sustainment (e.g., funding stability).

## Limitations

Our review was not intended to be systematic or scoping review of all sustainment measures. Rather, our purpose was to identify the currently available and accessible measures of sustainment for implementation researchers and practitioners. In doing so, we have identified several research considerations to advance pragmatic and psychometrically robust measurement of sustainment.

## Conclusion

There is a lack of pragmatic and psychometrically sound measures that can be completed by implementation stakeholders within inner context settings (e.g., frontline providers, supervisors). Further, there are a limited number of measures specifically addressing sustainment versus similar constructs of sustainability. Among these, current measures of sustainment are specific for particular settings or interventions, focus on outer context factors, and may be complicated for stakeholders to complete because of the outer context knowledge required. Our next steps are to address this need for a pragmatic measure of sustainment for inner context influencers that focuses on the integration and support perceived at a provider level.

## Data Availability

Not applicable

## References

[1] Rogers EM (2003). Diffusion of innovations.

[2] Graham ID, Logan J, Harrison MB (2006). Lost in knowledge translation: time for a map?. J Contin Educ Heal Prof.

[3] Kitson A, Rycroft-Malone J, Harvey G, McCormack B, Seers K, Titchen A (2008). Evaluating the successful implementation of evidence into practice using the PARiHS framework: theoretical and practical challenges. Implement Sci.

[4] Steckler A, Goodman RM, McLeroy KR, Davis S, Koch G (1992). Measuring the diffusion of innovative health promotion programs. Am J Health Promot.

[5] Aarons GA, Hurlburt M, Horwitz SM (2011). Advancing a conceptual model of evidence-based practice implementation in public service sectors. Adm Policy Ment Hlth.

[6] Fixsen DL, Naoom SF, Blase KA, Friedman RM, Wallace F (2005). Implementation research: a synthesis of the literature.

[7] Moullin JC, Dickson KS, Stadnick NA, Rabin B, Aarons GA (2019). Systematic review of the Exploration, Preparation, Implementation, Sustainment (EPIS) framework. Implement Sci.

[8] Wiltsey Stirman S, Kimberly J, Cook N, Calloway A, Castro F, Charns M. The sustainability of new programs and innovations: a review of the empirical literature and recommendations for future research. Implement Sci. 2012;7(17). 10.1186/1748-5908-7-17 PMC3317864 22417162

[9] Tricco AC, Ashoor HM, Cardoso R (2016). Sustainability of knowledge translation interventions in healthcare decision-making: a scoping review. Implement Sci.

[10] Proctor EK, Silmere H, Raghavan R (2011). Outcomes for implementation research: conceptual distinctions, measurement challenges, and research agenda. Adm Policy Ment Hlth.

[11] Rabin BA, Brownson RC, Haire-Joshu D, Kreuter MW, Weaver NL (2008). A glossary for dissemination and implementation research in health. J Public Health Manag Pract.

[12] Goodman RM, McLeroy KR, Steckler AB, Hoyle RH (1993). Development of level of institutionalization scales for health promotion programs. Health Educ Q.

[13] Pluye P, Potvin L, Denis JL, Pelletier J (2004). Program sustainability: focus on organizational routines. Health Promot Int.

[14] Finch TL, Rapley T, Girling M (2013). Improving the normalization of complex interventions: measure development based on Normalization Process Theory (NoMAD): Study protocol. Implement Sci.

[15] Slaghuis SS, Strating MMH, Bal RA, Nieboer AP. A framework and a measurement instrument for sustainability of work practices in long-term care. BMC Health Serv Res. 2011;11. 10.1186/1472-6963-11-314 PMC3234291 22087884

[16] Moullin JC, Sabater-Hernández D, Benrimoj SI (2016). Model for the evaluation of implementation programs and professional pharmacy services. Res Soc Adm Pharm.

[17] Bracht N, Finnegan JR, Rissel C (1994). Community ownership and program continuation following a health demonstration project. Health Educ Res.

[18] Glasgow RE, Vogt T, Boles S (1999). Evaluating the public health impact of health promotion interventions: The RE-AIM framework. Am J Public Health.

[19] Ament SMC, de Groot JJA, Maessen JMC, Dirksen CD, van der Weijden T, Kleijnen J. Sustainability of professionals’ adherence to clinical practice guidelines in medical care: a systematic review. BMJ Open. 2015;5(12). 10.1136/bmjopen-2015-008073 PMC4710818 26715477

[20] Shediac-Rizkallah MC, Bone LR (1998). Planning for the sustainability of community-based health programs: conceptual frameworks and future directions for research, practice and policy. Health Educ Res.

[21] Moore JE, Mascarenhas A, Bain J, Straus SE (2017). Developing a comprehensive definition of sustainability. Implement Sci.

[22] Gruen RL, Elliott JH, Nolan ML (2008). Sustainability science: an integrated approach for health-programme planning. Lancet..

[23] Aarons GA, Green AE, Willging CE, et al. Mixed-method study of a conceptual model of evidence-based intervention sustainment across multiple public-sector service settings. Implement Sci. 2014;9. 10.1186/s13012-014-0183-z PMC4272775 25490886

[24] Scheirer MA, Dearing JW (2011). An agenda for research on the sustainability of public health programs. Am J Public Health.

[25] Chambers DA, Glasgow RE, Stange KC (2013). The dynamic sustainability framework: addressing the paradox of sustainment amid ongoing change. Implement Sci.

[26] Scheirer MA (2005). Is sustainability possible? A review and commentary on empirical studies of program sustainability. Am J Eval.

[27] Shelton RC, Cooper BR, Stirman SW. The sustainability of evidence-based interventions and practices in public health and health care. Annu Rev Public Health. 2018. 10.1146/annurev-publhealth-040617-014731 29328872

[28] Proctor E, Luke D, Calhoun A, et al. Sustainability of evidence-based healthcare: research agenda, methodological advances, and infrastructure support. Implement Sci. 2015;10(88). 10.1186/s13012-015-0274-5 PMC4494699 26062907

[29] Iwelunmor J, Blackstone S, Veira D, et al. Toward the sustainability of health interventions implemented in sub-Saharan Africa: a systematic review and conceptual framework. Implement Sci. 2016;11. 10.1186/s13012-016-0392-8 PMC4804528 27005280

[30] Fleiszer AR, Semenic SE, Ritchie JA, Richer MC, Denis JL (2015). The sustainability of healthcare innovations: a concept analysis. J Adv Nurs.

[31] Society for Implementation Research Collaboration. The SIRC Instrument Review Project (IRP): a systematic review and synthesis of implementation science instruments. https://societyforimplementationresearchcollaboration.org/sirc-instrument-project/.

[32] GEM Grid-Enabled Measures Database: Sustainability. https://www.gem-measures.org/Public/ConstructDetail.aspx?cid=1198&cat=1, 2019.

[33] Penno LN, Davies B, Graham ID, et al. Identifying relevant concepts and factors for the sustainability of evidence-based practices within acute care contexts: a systematic review and theory analysis of selected sustainability frameworks. Implement Sci. 2019;14(1). 10.1186/s13012-019-0952-9 PMC6923954 31856861

[34] Kim MT, Han HR, Hedlin H (2011). Teletransmitted monitoring of blood pressure and bilingual nurse counseling–sustained improvements in blood pressure control during 12 months in hypertensive Korean Americans. The Journal of Clinical Hypertension.

[35] Shelton RC, Chambers DA, Glasgow RE. An extension of RE-AIM to enhance sustainability: addressing dynamic context and promoting health equity over time. Front Public Health. 2020;8. 10.3389/fpubh.2020.00134 PMC7235159 32478025

[36] Berta WB, Wagg A, Cranley L, et al. Sustainment, Sustainability, and Spread Study (SSaSSy): protocol for a study of factors that contribute to the sustainment, sustainability, and spread of practice changes introduced through an evidence-based quality-improvement intervention in Canadian nursing homes. Implement Sci. 2019;14(1). 10.1186/s13012-019-0959-2 PMC6923960 31856880

[37] Advanced Topics in Implementation Science (IS) Research Webinar Series [Internet]: National Cancer Institute; 2013 July 23, 2013. Podcast: 59:56.

[38] Palinkas LA, Spear SE, Mendon SJ (2020). Conceptualizing and measuring sustainability of prevention programs, policies, and practices. Transl Behav Med.

[39] Johnson AM, Moore JE, Chambers DA, Rup J, Dinyarian C, Straus SE (2019). How do researchers conceptualize and plan for the sustainability of their NIH R01 implementation projects?. Implement Sci.

[40] Stirman SW, Kimberly J, Cook N, Calloway A, Castro F. The sustainability of new programs and innovations: a review of the empirical literature and recommendations for future research. Implement Sci. 2012;7(17). 10.1186/1748-5908-7-17 PMC3317864 22417162

[41] Brown CH, Curran G, Palinkas LA (2017). An Overview of Research and Evaluation Designs for Dissemination and Implementation. Annu Rev Public Health.

[42] Aarons GA, Green AE, Trott E (2016). The roles of system and organizational leadership in system-wide evidence-based intervention sustainment: a mixed-method study. Adm Policy Ment Hlth.

[43] Lewis CC, Fischer S, Weiner B, Stanick C, Kim M, Martinez RG (2015). Outcomes for implementation science: an enhanced systematic review of instruments using evidence-based rating criteria. Implement Sci.

[44] Rabin BA, Purcell P, Naveed S (2012). Advancing the application, quality and harmonization of implementation science measures. Implement Sci.

[45] GEM-Dissemination and implementation initiative (GEM-D&I). http://www.gem-beta.org/GEM-DI, 2019.

[46] Damschroder L, Aron D, Keith R, Kirsh S, Alexander J, Lowery J (2009). Fostering implementation of health services research findings into practice: a consolidated framework for advancing implementation science. Implement Sci.

[47] Measures and Checklists. 2019; http://www.re-aim.org/resources-and-tools/measures-and-checklists/, 2019.

[48] Swain K, Whitley R, McHugo GJ, Drake RE (2010). The sustainability of evidence-based practices in routine mental health agencies. Community Ment Health J.

[49] Lubomski LH, Marsteller JA, Hsu Y-J, Goeschel CA, Holzmueller CG, Pronovost PJ (2008). The Team Checkup Tool: evaluating QI team activities and giving feedback to senior leaders. Jt Comm J Qual Patient Saf.

[50] Chamberlain P, Brown CH, Saldana L (2011). Observational measure of implementation progress in community based settings: the stages of implementation completion (SIC). Implement Sci.

[51] Maher L, Gustafson D, Evans A. NHS Sustainability Model and Guide. 2010.

[52] Luke DA, Calhoun A, Robichaux CB, Elliott MB, Moreland-Russell S. The program sustainability assessment tool: a new instrument for public health programs. Prev Chronic Dis. 2014;11. 10.5888/pcd11.130184 PMC3900326 24456645

[53] Clinical Sustainability Assessment Tool (CSAT). 2020; https://sustaintool.org/csat/, 2020.

[54] Mancini JA, Marek LI (2004). Sustaining community-based programs for families: conceptualization and measurement. Fam Relat.

[55] Thompson B, Lichtenstein E, Corbett K, Nettekoven L, Feng Z (2000). Durability of tobacco control efforts in the 22 Community Intervention Trial for Smoking Cessation (COMMIT) communities 2 years after the end of intervention. Health Educ Res.

[56] Bradley EH, Webster TR, Baker D, Schlesinger M, Inouye SK (2005). After adoption: sustaining the innovation a case study of disseminating the Hospital Elder Life Program. J Am Geriatr Soc.

[57] Sandoval JA, Lucero J, Oetzel J (2011). Process and outcome constructs for evaluating community-based participatory research projects: a matrix of existing measures. Health Educ Res.

[58] McIntosh K, MacKay LD, Hume AE (2011). Development and initial validation of a measure to assess factors related to sustainability of school-wide positive behavior support. J Posit Behav Interv.

[59] Amodeo M, Storti SA, Larson MJ (2010). Moving empirically-supported treatment to the workplace: recruiting addiction program supervisors to help in technology transfer. Subst Use Misuse.

[60] Solberg LI, Asche SE, Margolis KL, Whitebird RR (2008). Measuring an organization's ability to manage change: the change process capability questionnaire and its use for improving depression care. Am J Med Qual.

[61] Eisen JC, Marko-Holguin M, Fogel J, et al. Pilot study of implementation of an internet-based depression prevention intervention (CATCH-IT) for adolescents in 12 US primary care practices: clinical and management/organizational behavioral perspectives. The Primary Care Companion for CNS Disorders. 2013;15(6). 10.4088/PCC.10m01065 PMC3977759 24800110

[62] Skinner K (2007). Developing a tool to measure knowledge exchange outcomes. Can J Program Eval.

[63] Berliner L. Organization Checklist. Available from: https://societyforimplementationresearchcollaboration.org/sustainability-8/.

[64] Stamatakis KA, McQueen A, Filler C (2012). Measurement properties of a novel survey to assess stages of organizational readiness for evidence-based interventions in community chronic disease prevention settings. Implement Sci.

[65] Chung H, Duffy FF, Katzelnick DJ (2013). Sustaining practice change one year after completion of the national depression management leadership initiative. Psychiatr Serv.

[66] Roundfield KD, Lang JM (2017). Costs to community mental health agencies to sustain an evidence-based practice. Psychiatr Serv.

[67] Bond GR, Drake RE, Rapp CA, McHugo GJ, Xie H (2009). Individualization and quality improvement: two new scales to complement measurement of program fidelity. Adm Policy Ment Hlth.

[68] Gaglio B, Shoup JA, Glasgow RE (2013). The RE-AIM framework: a systematic review of use over time. Am J Public Health.

[69] Chan KS, Hsu Y-J, Lubomski LH, Marsteller JA (2011). Validity and usefulness of members reports of implementation progress in a quality improvement initiative: findings from the Team Check-up Tool (TCT). Implement Sci.

[70] Marsteller JA, Hsu Y-J, Chan KS, Lubomski LH (2017). Assessing content validity and user perspectives on the Team Check-up Tool: expert survey and user focus groups. BMJ Qual Saf.

[71] Yin RK (1981). Life histories of innovations: how new practices become routinized. Public Adm Rev.

[72] Finch TL, Girling M, May CR (2018). Improving the normalization of complex interventions: part 2-validation of the NoMAD instrument for assessing implementation work based on Normalization Process Theory (NPT). BMC Med Res Methodol.

[73] Glasgow RE, Riley WT (2013). Pragmatic measures: what they are and why we need them. Am J Prev Med.

[74] Novins DK, Green AE, Legha RK, Aarons GA (2013). Dissemination and implementation of evidence-based practices for child and adolescent mental health: a systematic review. J Am Acad Child Adolesc Psychiatry.

[75] Hunter SB, Han B, Slaughter ME, Godley SH, Garner BR (2017). Predicting evidence-based treatment sustainment: results from a longitudinal study of the Adolescent-Community Reinforcement Approach. Implement Sci.

[76] Schell SF, Luke DA, Schooley MW (2013). Public health program capacity for sustainability: a new framework. Implement Sci.

[77] Feldstein A, Glasgow R (2008). A practical, robust implementation and sustainability model (PRISM). Jt Comm J Qual Patient Saf.

[78] Herlitz L, MacIntyre H, Osborn T, Bonell C. The sustainability of public health interventions in schools: a systematic review. Implement Sci. 2020;15(4). 10.1186/s13012-019-0961-8 PMC6945701 31906983

[79] Lennox L, Maher L, Reed J. Navigating the sustainability landscape: a systematic review of sustainability approaches in healthcare. Implement Sci. 2018;13(1). 10.1186/s13012-017-0707-4 PMC5810192 29426341

[80] Bruns EJ, Kerns SE, Pullmann MD, Hensley SW, Lutterman T, Hoagwood KE (2016). Research, data, and evidence-based treatment use in state behavioral health systems, 2001-2012. Psychiatr Serv.

[81] McHugh RK, Barlow DH (2010). The dissemination and implementation of evidence-based psychological treatments: a review of current efforts. Am Psychol.

[82] Lewis CC, Mettert KD, Dorsey CN, et al. An updated protocol for a systematic review of implementation-related measures. Implement Sci. 2017;12. 10.1186/s13643-018-0728-3 PMC5918558 29695295

